# A Clinical AI-Based System (MoodMon) for Affective Disorders: Algorithm Development and Validation

**DOI:** 10.2196/89981

**Published:** 2026-09-02

**Authors:** Marlena Sokół-Szawłowska, Łukasz Święcicki, Katarzyna Kolasa, Katarzyna Kaczmarek-Majer

**Affiliations:** 1 Psychiatric Outpatient Clinic, Institute of Psychiatry and Neurology, Sobieskiego 9, Warsaw, Mazovia, 02-957, Poland, 48 604217639; 2 II Clinic of Psychiatry, Institute of Psychiatry and Neurology, Warsaw, Mazovia, Poland; 3 Department of Economics, Kozminski University, Warsaw, Poland; 4 Systems Research Institute, Polish Academy of Sciences, Warsaw, Poland

**Keywords:** bipolar disorder, depression, AI, biomarkers, physical voice parameters, mental health monitoring, mobile app, artificial intelligence

## Abstract

**Background:**

Psychiatry needs objective technological tools to address global staffing shortages, stigma, and other systemic challenges. An AI-based system for mood monitoring (MoodMon) was developed along with a mobile app for smartphones to detect changes in the mental state of individuals with major depressive disorder (MDD) and bipolar disorder (BD) based on acoustic features derived from speech signals. A long-term, naturalistic study of the MoodMon system represents a breakthrough in biomarker validation.

**Objective:**

The aim of the study was to determine whether acoustic features are effective biomarkers of mental status changes in individuals with affective disorders and whether they are useful in the remote clinical monitoring of patients by psychiatrists.

**Methods:**

To evaluate the effectiveness of AI algorithms in detecting changes in mental state based on acoustic features, data from 75 patients with BD and 25 patients with MDD over a period of 944 days were used. This makes this the longest analysis in the world covering two of the most common mental disorder diagnoses. A wealth of clinical, behavioral, and technical data were collected and used to train the MoodMon machine learning models under the supervision of human experts—experienced psychiatrists. In the first stage, the AI was trained using objective data and clinical assessments conducted by psychiatrists, including 17-item version of the Hamilton Depression Rating Scale and the Young Mania Rating Scale, as well as the Clinical Global Impression Scale. The second stage involved further refinement of the AI models using individual and population data, generating alerts when subtle changes in mental state were detected.

**Results:**

In total, 243 acoustic features were extracted from the speech signals of patients and considered as input for the AI models aimed at detecting changes in mental state. The system demonstrated high performance, achieving the following sensitivity (true positive rate [TPR]) and specificity (true negative rate [TNR]) values: for both diagnoses, a TPR of 89.5% (1151/1286) and a TNR of 98.8% (30,224/30,579); for BD only, a TPR of 89.6% (866/966) and a TNR of 98.9% (22,664/22,905); and for MDD only, a TPR of 89.1% (285/320) and a TNR of 98.5% (7560/7674). Voice alerts in the MoodMon system are a key tool supporting clinical decision-making. They increase the probability of a clinical visit and exert a significant influence on the likelihood of treatment modification.

**Conclusions:**

The system confirmed the presence of parameters that may serve as biomarkers of mental state changes in individuals with BD and MDD. A key clinical implication is the increased probability of prompt treatment modification following an alert, thereby supporting the primary objective underlying the development of the AI-based MoodMon system.

## Introduction

Recent systematic reviews indicate that the integration of digital biomarkers derived from ubiquitous consumer devices (eg, smartphones and wearables) into the diagnosis, treatment, and longitudinal monitoring of mood disorders constitutes a highly promising paradigm. When combined with the capacity of AI and machine learning algorithms to process large-scale, high-dimensional, and temporally continuous datasets, these approaches enable the extraction of clinically meaningful patterns that are otherwise inaccessible through conventional assessment methods. Consequently, such technologies offer substantial potential for the implementation of personalized and adaptive monitoring and therapeutic strategies in mood disorders, with an additional prospective benefit of improving adherence to pharmacological treatments. Nevertheless, the primary objective of deploying AI-driven solutions in mental health care extends beyond diagnostic optimization and treatment compliance. The overarching goal remains the identification of scalable, evidence-based strategies that effectively enhance the quality of life and overall psychosocial functioning of patients [1].

Despite advances in clinical psychiatry and digital health technologies, the diagnosis and management of mood disorders, including major depressive disorder (MDD), remain intrinsically complex. Key barriers include limited access to specialized mental health services and persistently low long-term treatment adherence, which are strongly influenced by sociodemographic and systemic factors [2,3]. Notably, even in high-income countries, only approximately 50% of individuals with MDD receive a formal diagnosis and adequate follow-up care. On a global scale, this proportion decreases to nearly 30% in low- and middle-income countries, where additional obstacles, such as stigma related to mental health, further restrict access to care [4,5].

The urgency of addressing these challenges is underscored by the high global burden of affective disorders, which—alongside anxiety disorders—represent the most prevalent categories of mental health conditions worldwide. Bipolar disorder (BD) is a chronic, recurrent affective illness characterized by marked fluctuations in mood, cognition, behavior, psychomotor activity, and functional capacity, frequently leading to substantial impairments in interpersonal relationships and occupational functioning. The estimated lifetime prevalence of BD ranges from 0.4% to 1.1% globally [6,7].

Accurate diagnosis of BD remains a major clinical challenge due to its heterogeneous presentation, variable course, and symptom overlap with other psychiatric conditions, particularly unipolar depression. These factors contribute to high rates of misdiagnosis and significant delays in the initiation of appropriate treatment [3,8,9].

Depressive disorders represent one of the leading causes of disability worldwide, affecting an estimated 300 million individuals [10-12]. Beyond the profound psychological burden, depression is associated with substantial impairments in social functioning, including the inability to fulfill age-appropriate social and occupational roles. Moreover, depression is frequently comorbid with somatic conditions, particularly metabolic disorders, which are often inadequately treated as a consequence of depressive symptomatology itself [13-16].

## Methods

### Study Design

The MoodMon system was developed by an interdisciplinary team. The study was conducted between 2021 and 2023. The analytical engine (AI) was trained on data collected from patients over a study period of 944 days. A total of 100 patients participated: 75 patients with BD (type I or II) and 25 patients with recurrent depression. Informed consent was required. A longitudinal, naturalistic study was designed. Patients were recruited while undergoing standard treatment (in accordance with Polish pharmacological and psychological recommendations) in 8 outpatient clinics in Warsaw and Poznań, Poland.

### Ethical Considerations

The Bioethics Committee at the District Medical Chamber approved the study (approval number: KB 170/2021; extension number: 366/2021). Patients provided their initial informed consent to participate in the study, which allowed for analysis without requiring additional consent for secondary analyses of the research data. Privacy and confidentiality were protected by anonymizing the research data. Participants did not receive any compensation for their participation.

### Recruitment

Only individuals at high risk of relapse were recruited. The aim of this procedure was to enable the potential observation of a large number of changes in the natural environment of standard outpatient psychiatric treatment. Inclusion criteria were at least 2 phase transitions in the previous 14 months for patients with BD and at least 1 episode during this period for patients with recurrent depression. After the first (observational) phase, 12 patients withdrew from the study, with 88 patients remaining: 66 with BD and 22 with recurrent depression. Eighty-four patients (BD: n=64; MDD: n=20) participated in the study for over 18 months.

### Tested AI System

The MoodMon system includes (1) a mobile app for patients (for Android smartphones) that collects and transmits data to secure servers; (2) an AI-based analytical engine that processes the collected data and learns to send alerts based on objective device data, including acoustic features and psychiatrists’ clinical assessments; and (3) a web portal for physicians that collects demographic data, visit data, and physician reviews and visualizes observation results.

In the study’s initial phase, researchers evaluated the mental states of patients during every interaction, recording the results via the web portal. These evaluations served as ground-truth labels for the corresponding voice samples used to train the model. During the second phase, the AI engine generated automated alerts based on patient voice data. These notifications were sent to the patient, their caretaker (if designated), and the clinician’s web portal, triggering a psychiatric consultation. Subsequently, the portal facilitated a formal validation of the clinical justification for each alert.

**Figure 1.**
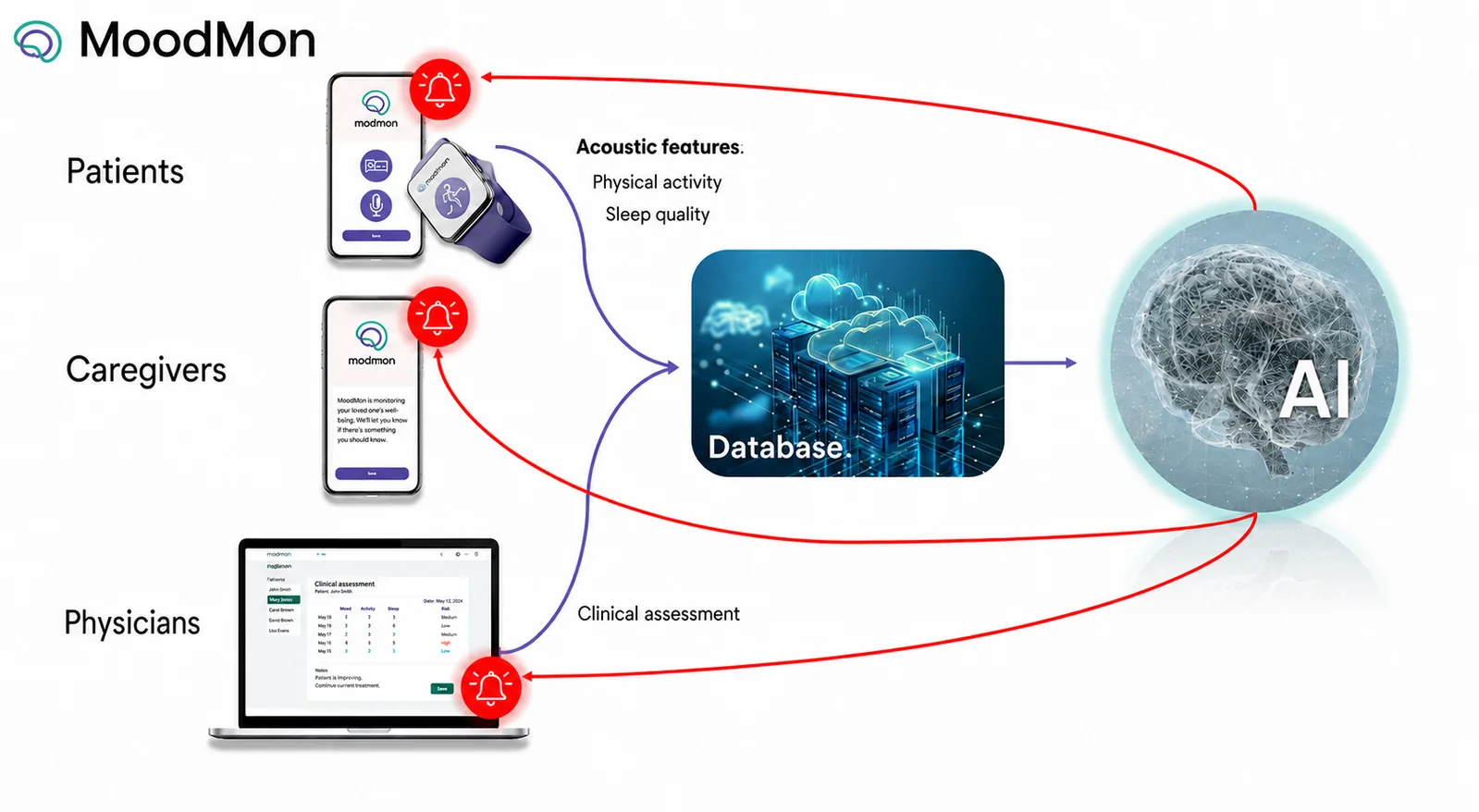
Main functions and components of the MoodMon system.

The MoodMon system is schematically illustrated in Figure 1.

#### Acoustic Features From Speech and Behavioral Data Collection

Participants were asked to complete a daily voice survey consisting of responses to three randomly selected questions from a list of 85, which did not refer to the disease or other sensitive topics. Voice recordings were collected each day. Mental state assessments were conducted by 8 independent psychiatric researchers, none of whom had access to the voice recordings, and individuals with the condition were blinded to the speech signal processing process. Voice recordings were processed using an automated signal processing pipeline that extracted standard acoustic features. Behavioral data were continuously collected via smartphones, whereas clinical assessments occurred at substantially lower temporal resolution. Since supervised training of the AI model required temporal alignment of both data modalities, psychiatric labels were extrapolated across intervals without direct clinical assessment.

Specifically, if no change in the CGI score was observed between 2 consecutive assessments conducted 2 weeks apart, all intervening days were assigned the same CGI label. For the days between 2 assessments with different CGI scores, the logic generated two probabilistic entries per day, weighted by 1/N (change) and (N-1)/N (no change), to account for the temporal uncertainty of the transition. Periods missing an initial or final score were excluded from the training dataset.

The validation was performed on the test dataset, which was separate from the training one.

#### Data Preprocessing

Datasets were considered complete when all parameters were available; partial datasets were supplemented when one or more daily parameters were missing. Missing values of observed parameters were imputed using the mean of the observations over the preceding 14 days, provided that no more than 7 days of data were missing within that interval. When such an average could not be reliably computed, the corresponding dataset was excluded from further analysis. Values to supplement the missing data were calculated over 14-day periods only, as longer periods could cover different patient conditions and make the imputed values unreliable.

#### Baseline Data Protocol

According to the protocol, historical clinical data from the 2 years preceding the study, such as the number of disease episodes and the number of hospitalizations of patients, came from the original medical records of the patients and were entered into the electronic documentation of the project.

### Detailed Description of the AI Algorithm and Acoustic Features Selection

Data collected in the first phase of the study allowed for the identification of parameters with the highest predictive power. Based on interviews with specialist physicians and the literature, 243 parameters with predictive potential were identified. In the first stage of narrowing down this list, we eliminated those whose collection was difficult or expensive for technical reasons, or inadvisable due to the need to maintain patient privacy and comply with personal data regulations. To analyze the stability of the obtained results, a bootstrap analysis was performed, and a list of 50 variables with the highest weights was selected. This analysis revealed that acoustic features were the most variable-monitored attributes. The final feature selection was performed using principal component analysis, applying the criterion of selecting the number of principal components at 90% of the cumulative variance of the explained variable (patient condition variable). As a result of feature engineering and selection processes, a subset of 19 acoustic features was selected for the decision-making module, including fundamental frequency; selected mel-frequency cepstral coefficients; energy within predefined frequency bands; pause counts and durations; and voice quality features, for example, jitter and shimmer. Our analysis confirmed previous reports, which indicated the variability of fundamental frequency and mel-frequency cepstral coefficients as the acoustic features most strongly associated with emotional states in the course of affective disorders [17-20].

### Study Phases and Validation

In this study, we included individuals with documented transitions to ensure that the model’s performance reflects its predictive capability in clinically dynamic and real-life scenarios.

Given the extremely individual course of affective disorders, it was decided that comparison with a different group of patients was not adequate [21,22]. In affective disorders, the patient in the euthymic state serves as a reference for themselves. For this reason, the study was a single-arm study without a control group.

### Phase I: Data Acquisition and Model Pretraining

#### Overview

The first phase comprised a 12-month observation period for each participant. Its primary objectives were as follows: (1) to collect multimodal data for evaluating their predictive use and (2) to obtain a dataset of sufficient size and diversity for the initial training of the AI models. In the initial phase of the experimental evaluation, we considered the top-performing benchmark machine learning algorithms, including random forest, decision trees, support vector classifier, recurrent neural network, logistic regression, and light gradient boosting machine. For variable selection, the recursive feature elimination and Boruta algorithms were considered. Although full transparency of gradient boosting algorithms, such as light gradient boosting machine, cannot be achieved, we acknowledge interpretability approaches, for example, Shapley additive explanations–based methods, that enable us to gain further insights into the model’s behavior.

The final decision of the system was the result of the logical product of both algorithms, that is, the system alerted about a change if both algorithms predicted the change. At baseline, an in-person visit was conducted to collect demographic and clinical information. The current mental state was assessed using the 17-item Hamilton Depression Rating Scale (HDRS) and the Young Mania Rating Scale (YMRS). Symptom severity was additionally quantified using a version of the Clinical Global Impression Scale (CGI) adapted to the study protocol. Subsequent clinical assessments were scheduled at 3-month intervals. Due to the COVID-19 pandemic, both in-person and remote visits were permitted. At each visit, HDRS and YMRS scores constituted the primary outcome measures for symptom severity evaluation. Previous works, aimed at identifying early and subtle indicators of affective phase transitions, suggest the following diagnostic cutoffs: euthymia (HDRS<8 and YMRS<6), depression (HDRS≥8 and YMRS<6), hypomania or mania (HDRS<8 and YMRS≥6), and mixed state (HDRS ≥8 and YMRS≥6) [23,24]. In this study, following each clinical contact, the psychiatrist—based on examination and clinical expertise—assigned 1 of 7 affective states: mania, hypomania, euthymia (balanced mood), subthreshold depression, depression, severe depression, or mixed state. For analytical purposes, this expert assessment was encoded as a CGI value and used as a supervisory label for the behavioral and acoustic features collected on the corresponding day.

In addition to scheduled visits, every 2 weeks a psychiatrist evaluated the mental state of the patient based on structured telephone interviews, focusing on potential changes in mood and psychomotor activity. If the questionnaire results suggested a clinically relevant change, the patient was invited for an intervention visit involving a full clinical assessment using standardized rating scales.

#### Feature Selection and Engineering

The categorical variable with diagnosis (BD vs MDD) was considered in the initial exploratory analyses. We performed a weight of evidence analysis to determine the initial set of categorical features for further analyses. Next, secondary bootstrap analyses and principal component analysis were performed, including both continuous and categorical variables. During the course of the experiments, and considering the limited sample size while aiming to avoid overfitting, the final subset of features considered only selected acoustic features that proved to be the most powerful. Neither diagnosis (BD vs MDD) nor gender proved to be more significant than acoustic features for inclusion in the final predictive modeling.

### Phase II: Model Deployment and Prospective Evaluation

#### Overview

In the second phase, the pretrained AI module analyzed incoming acoustic features on a daily basis to determine whether they indicated an imminent change in the mental state of the patient. Upon detection of such a change, the system generated an alert, which was visualized for the clinician via the MoodMon physician portal. Importantly, the system did not infer the direction of the detected change (eg, depressive vs manic shift); it solely indicated the presence of a significant deviation from the patient’s baseline pattern. The system was also capable of detecting changes potentially unrelated to the underlying affective disorder, which might act as triggers for clinical episodes occurring in the prodrome stage. The inclusion of these factors in this study is well-supported by the current literature for both diagnoses [25,26].

A key innovation of the MoodMon system was its real-time alerting mechanism, which signaled potential shifts in mental state. This enabled clinicians to perform comprehensive assessments at the onset of an episode, allowing for timely intervention or informed observation. Crucially, the system maintained a “human-in-the-loop” approach: the final clinical judgment and therapeutic decisions rested solely on the physician’s subjective expertise, ensuring AI recommendations were filtered through professional experience.

#### Clinical Assessment

In the second phase of the project, visits took place at 3-month intervals and in response to an alert sent to the physician by the MoodMon system. Short telephone visits followed the alert, or if clinically necessary, longer in-person visits. Data collection continued as in the first phase. The predictive model was continuously and systematically trained, and after collecting sufficient data for each patient, individualized predictions were enabled by applying an individual decision-making threshold for the alert. The validity of each alert was assessed by the psychiatrist based on the patient’s description of the change or lack thereof in their mental state, taking into account important life circumstances. Each alert received 1 of 4 possible ratings: justified, unjustified, justified by other circumstances, and impossible to assess. Each alert also provided a CGI rating of the patient’s condition on the day of the alert. Treatment modifications in the clinical trial included changing the drug dosage, adding medications, changing medications, discontinuing medications, adding psychotherapy, and issuing a referral to a hospital. To reduce confirmation bias during alert assessment, the project used procedures consisting of (1) initial training on hypothetical clinical situations for a team of psychiatrists and (2) implementation of systematic meetings of psychiatrists (every 2 wk) throughout the first and second stages of the study, with ongoing supervision over the standardization of model training and alert validation.

#### System Validation

The assessments of the validity of the alerts provided by the psychiatrists were used to calculate model quality measures, specifically the true positive rate (TPR; sensitivity) and the true negative rate (TNR; specificity). TPR is the ratio of correctly predicted mood changes.

$TPR=\frac{\Sigma TP}{\Sigma TP+\Sigma FN}$(1)

TNR is the ratio of correctly predicted “no changes.”

$TNR=\frac{\Sigma TN}{\Sigma TN+\Sigma FP}$(2)

True and false positives are defined in the following way: (1) true positive (TP) is denoted when an alert is issued and assessed as justified (a mood change occurred since the patient’s previous clinical assessment); the alert about a change in the patient’s mood, assessed as justified by factors other than an affective disorder, was labeled TP, as a circumstance requiring attention and exclusion of the upcoming episode; (2) true negative (TN) is denoted when no alert is issued and its absence is assessed as correct (no mood change since the previous clinical assessment of the patient); (3) false positive (FP) is denoted when an alert is sent but assessed as unjustified (no mood change since the patient’s previous clinical assessment); and (4) false negative (FN) is denoted when no alert is issued and this situation is assessed as incorrect (a mood change occurred since the patient’s previous clinical assessment).

To calculate the above measures, each decision made by the system to send an alert or not was evaluated; that is, for each day of each patient participating in the second stage of the study, 1 of the 4 possible labels (TP, FP, TN, and FN) was selected. Technological details are provided in a separate publication [27].

### Alert-to-Action Timelines Analysis

All clinical data and alert evaluations were integrated into a single analytical database. Basic descriptive statistics were used to characterize the dataset and evaluate alert performance. Continuous variables (eg, time from alert to visit, time to treatment modification, and acoustic parameters) were summarized using mean and median. Event-based metrics, such as the number of alerts, visits, and treatment changes, were reported as sums and proportions. Alert-to-action timelines were analyzed by calculating mean and median intervals for visits occurring within 48 hours, between 48 hours and 7 days, and beyond 7 days. These descriptive measures provided an initial assessment of system responsiveness and the clinical relevance of AI-generated alerts.

## Results

The average duration of the disease among the study participants: 8.6 years for patients with BD and 8 years for patients with MDD. Other demographic data are provided in Table 1.

**Table 1.** Demographics of the population of patients using the MoodMon system in the study.

Category	MDD^a^ (n=25), n (%)	BD^b^ (n=75), n (%)^c^
Age range (y)		
18‐24	6 (24)	6 (8)
25‐34	6 (24)	18 (24)
35‐44	6 (24)	20 (27)
45‐54	6 (24)	16 (21)
55‐64	1 (4)	15 (20)
Gender		
Men	7 (28)	33 (44)
Women	18 (72)	42 (56)
Marital status		
Divorced	2 (8)	11 (15)
Single	13 (52)	35 (47)
Married	10 (40)	27 (36)
Widow	0 (0)	2 (3)
Education		
Higher	13 (52)	56 (75)
Secondary	11 (44)	18 (24)
Vocational	1 (4)	1 (1)
Professional activity		
Employed	17 (68)	52 (69)
Other	3 (12)	4 (5)
Pensionist	1 (4)	10 (13)
Student	4 (16)	7 (9)
Unemployed	0 (0)	2 (3)
Type of household		
Alone	6 (24)	18 (24)
With a partner	3 (12)	13 (17)
With family	16 (64)	44 (59)
Town size		
>200,000	15 (60)	57 (76)
5000‐200,000	5 (20)	13 (17)
<5000	5 (20)	5 (7)

a MDD: major depressive disorder.

b BD: bipolar disorder.

c Percentage may not sum to 100% due to rounding.

### Phase I and II Results

Throughout the study period, there were 24,258 single instances of system use for patients with BD and 7636 for individuals with MDD. A total of 1394 changes in patients’ mental states were documented. A total of 97,428 behavioral datasets were collected, of which 53,934 were complete and accompanied by a corresponding clinical evaluation of the patient’s condition on the same day. Among these, 1344 affective state transitions were identified with full clinical and behavioral correspondence, forming the basis for the training of AI models. During the study, 4570 phases were captured and assigned to 7 distinct affective states. The following clinical activities were conducted: 100 baseline visits, 410 in-person follow-up visits, 1908 telephone assessments, 760 intervention visits, and 100 final evaluations.

In phase II, a total of 2656 automated alerts were generated, of which 1587 were subsequently reviewed and verified by psychiatrists.

### Validation Results

The counts of TP, TN, FP, and FN labels obtained in the second phase of the study are as follows: there were 1151 TPs, 355 FPs, 30,224 TNs, and 135 FNs.

The overall system performance, calculated across both diagnostic groups, demonstrated a TPR of 89.5% (1151/1286) and a TNR of 98.8% (30,224/30,579). For patients with BD, the model achieved a TPR of 89.6% (866/966) and a TNR of 98.9% (22,664/22,905), whereas for individuals with MDD, the results were a TPR of 89.1% (285/320) and a TNR of 98.5% (7560/7674). The precision of the system was as follows: a positive predictive value of 76.4% and an *F*_1_-score of 0.824.

### Alert-to-Action Timelines Results

Phase II lasted 16 months, during which system alerts were sent to clinicians for evaluation and to patients and caregivers where applicable. To avoid alert fatigue and patient anxiety, a 5-day suppression window was applied; alerts generated within 5 days of a primary notification were withheld as duplicate representations of the same clinical change. Summary statistics for generated, sent, and assessed alerts are presented in Table 2.

During the second phase of the study, scheduled care adhered to standard clinical treatment protocols for affective disorders, consisting of quarterly in-person consultations. Unscheduled visits were arranged upon patient request, and system-generated alerts were intended to prompt contact between the patient and psychiatrist. Table 3 summarizes the breakdown of alert-triggered visits, follow-up consultations indirectly related to alerts, and visits not related to alerts.

The high proportion of alert-driven visits (combined alert-triggered and follow-up consultations)—accounting for 61% (1069/1754) of all encounters—indicates that the alert system functioned as a major driver of patient-clinician contact.

Categorization by contact type reveals that telephone consultation was frequently chosen as postalert communication modality (Table 4).

Temporal stratification of postalert consultations revealed a distinct pattern. The distribution of visits categorized by alert-to-contact interval is detailed in Table 5.

Mean and median time intervals were calculated for each stratum. These metrics, presented in Table 6, provide insight into the operational responsiveness of the AI-supported monitoring system.

Automated alerts facilitated earlier and more frequent treatment modifications following clinical changes in patient mental state. Compared with non–alert-related encounters, alert-triggered visits exhibited a higher proportion of treatment adjustments (Table 7) as well as a significantly higher modification frequency (Table 8).

These findings underscore the clinical utility of AI-generated alerts in psychiatric management. By translating continuous monitoring into timely patient-clinician engagement, the system facilitates dynamic, data-driven therapeutic adjustments, enabling proactive interventions prior to significant clinical deterioration. Integrating AI-driven alerts into routine clinical workflows bridges the gap between scheduled visits and acute patient needs. This approach transforms passive monitoring into actionable clinical decisions, laying the foundation for more responsive and individualized care in affective disorders.

**Table 2.** Number of alerts generated, suppressed, and evaluated.

Alert action type	Count, n
Alerts generated	2656
Alerts sent for evaluation	1705
Alerts assessed	1588

**Table 3.** Breakdown of visits carried out in the second phase of the study.

Visit type	Count (N=1754), n (%)
Visits directly related to alerts	982 (56)
Follow-up visits (indirectly related to alert)	87 (5)
Visits not related to alerts	685 (39)
Total visits	1754 (100)

**Table 4.** Breakdown of alert-triggered visits by contact type.

Contact type	Count, n
In person	448
Phone contact	534

**Table 5.** Temporal stratification of alert-driven visits.

Temporal strata	Count, n
Up to 48 h	720
Between 3 and 7 days	194
Between 8 to 14 d	68

**Table 6.** Mean and median alert-to-action intervals by temporal strata.

Alert-to-visit interval	Medium interval (SD)	Medium interval (IQR)
Up to 48 h	0.85 (0.71)	1 (0-1)
Between 3 and 7 days	4.31 (1.35)	4 (3-5.75)
Between 8 to 14 d	10.25 (2.03)	10 (8-12)

**Table 7.** Number of treatment modifications in visits related to alerts and those not related.

Relation of alert with treatment modification	Total, N	Resulting in treatment modification, n (%)
Visits related to alert	982	141 (14)
Visits not related to alert	772	78 (10)

**Table 8.** Consecutive treatment modifications intervals.

Patient-clinician contact interval	Consecutive treatment modifications interval	
	Mean (SD)	Median (IQR)
Visits up to 48 h after alert	36.69 (54.98)	19 (5-41)
Visits between 3 and 7 days after alert	30.77 (34.18)	14 (5-36)
Visits between 8 to 14 d after alert	23.83 (14.78)	15.5 (7.88-23.13)
Visits not related to alert	72.71 (93.26)	30.5 (6.15-100.15)

## Discussion

### Main Results

The MoodMon system demonstrated technical capability in distinguishing affective states in individuals with BD using acoustic features [27]. Notably, monitoring dynamic changes in mental state based on a limited set of acoustic features appears particularly effective in the very early or prodromal phase of affective episodes. Through real-time alert generation, both patients and their treating psychiatrists gained rapid access to objective information regarding potential changes in mental status. When such alerts were subsequently confirmed as clinically relevant during psychiatric evaluation, therapeutic interventions could be initiated earlier than would typically be possible under standard organizational conditions. By enabling continuous, objective monitoring beyond scheduled clinical visits, MoodMon facilitates greater continuity of care for patients with affective disorders. The present findings support prior theoretical and empirical work suggesting that continuous analysis of vocal biomarkers may substantially enhance the early detection of affective state changes, particularly during prodromal stages, thereby introducing novel clinical value [20,28-31]. Although behavioral markers were also collected during the study, their correlation with clinician-confirmed changes in mental state was weaker than that observed for acoustic features. High alert performance was achieved using the analysis of a limited number of acoustic parameters, indicating that effective monitoring does not require complex or resource-intensive sensing modalities. Consequently, simpler, cost-efficient, and widely accessible solutions can be used, as patients are not required to wear dedicated monitoring devices; instead, natural speech recorded via standard smartphones is sufficient. These findings align with existing literature demonstrating the high potential of objective acoustic features in longitudinal patient monitoring [20,32].

The model achieved high sensitivity and specificity (>89% and>98%, respectively) by adopting a binary classification approach (detecting “change” vs “no change”). This simplified framework, supported by previous research [27] and clinical consultation, met the practical requirements of practitioners who did not require more granular data. Furthermore, this binary design helped minimize potential confirmation bias during the evaluation.

The other important factor increasing the system’s effectiveness was continuous training and adjustments to the decision threshold for individual patients.

The dataset used for the system assessment was dominated by stable days, as is expected in an outpatient setting and corresponds to the intended use of the device. The model demonstrated high sensitivity and robust precision. Given the inherent class imbalance, precision serves as a more reliable performance indicator than accuracy [31]. The results confirm that the model remains highly effective despite the low prevalence of TP cases and that the risk of “alert fatigue” is relatively low. Additionally, it should be mentioned that in stable outpatient monitoring, the dominance of TN cases reflects the intended use and is not a methodological artifact.

The methodological framework of the MoodMon system adheres to contemporary recommendations for machine learning–based voice biomarker development, which emphasize the central role of human oversight in AI-assisted clinical systems [33]. In MoodMon, alerts generated during the second phase of the study underwent clinical validation by psychiatrists with relevant expertise, supported by standardized assessment scales. Critically, final clinical decisions remained exclusively the responsibility of the physician. The AI system was limited to detecting statistically significant deviations within the analyzed data streams, while the psychiatrist determined whether a clinical consultation was warranted and, if so, whether therapeutic modifications were necessary. This design ensures patient safety and preserves the primacy of clinical judgment, consistent with ethical and practical recommendations for the application of AI in psychiatry [33,34].

Just over 9% of alerts were assessed more than 7 days after their occurrence. The most common factors contributing to this were difficulties in contacting patients (during or after the COVID-19 pandemic). The psychiatrist or researcher always had the option to select “alert unassessable” if they were clinically unsure, for example, because the assessment was too far removed from the alert. Additionally, in the second phase of the study, scheduled visit slots could be used to assess alerts to avoid excessive involvement of patients at short intervals.

A deliberate design choice is for the AI to detect deviations from the baseline without identifying the direction (eg, depressive vs manic) and to alert users to factors documented in clinical studies as triggers for an episode (eg, psychosocial stress and somatic illness). For psychiatrists, who have previously relied on often-delayed retrospective reports from the patient with a psychiatric condition and/or their loved ones, this provides an opportunity to identify triggers or subtle initial (prodromal) symptoms earlier. In this context, transient mood swings signaled by alerts are equally clinically important for psychiatrists, as they practically represent, for example, insufficient efficacy of medications. The primary goal in the long-term treatment of affective disorders is achieving full remission (symptomatic and functional).

Clinical use of the MoodMon system demonstrated that AI-generated alerts constitute a meaningful support tool in psychiatric decision-making. Alerts not only increased the probability of follow-up visits for patients but also significantly influenced the likelihood of treatment modification. This represents a key clinical implication, confirming that the interdisciplinary objective underlying the development of MoodMon—namely, the creation of a clinically actionable, AI-supported monitoring system for patients and psychiatrists—has been achieved.

The clinical safety of the studied AI system was determined by the absence of adverse events associated with the MoodMon app. While not a primary end point, self-reported data—inclusive of dropouts—revealed a shift from 12 hospitalizations in the 2 years preceding the study to zero upon its conclusion, providing a compelling signal of the system’s clinical safety and use.

### Limitations

The system evaluated in this study was specifically designed for the remote monitoring of outpatients, and accordingly, the study population consisted exclusively of outpatient individuals. As a consequence, a disproportionately high number of TN cases was observed, corresponding to days without clinically relevant changes in the condition of the patient and without system-generated alerts. Therefore, the observed class distribution reflects real-world clinical conditions rather than methodological bias. Importantly, the results demonstrate genuinely high specificity, which is a critical prerequisite for clinical applicability; insufficient specificity would result in excessive false alerts and, consequently, limited clinical utility of the device.

The limitation of our naturalistic study is the omission of the influence of possible side effects of the medications used on voice parameters, although the outpatients did not receive very strong treatments typically used for severe conditions treated in closed wards of psychiatric hospitals in our country.

The researchers were aware that the standard practice of supplementing data with averages from periods preceding clinical assessments was applied, and that such a procedure could mask the rapid or ultrarapid changes in the mental state of a patient with affective disorders. An important consideration for in-depth analysis is that when the psychiatrist obtained information from the patient on this topic during the study, he placed it in the appropriate place in the system to provide a complete picture of the psychopathology.

Due to budgetary constraints, the mobile app used for voice sample acquisition was implemented exclusively for smartphones running the Android operating system (version 9 or later). While this restricted the rate of participant recruitment and introduced a degree of demographic stratification attributable to the correlation between smartphone operating systems and socioeconomic status, it did not affect the validity or quality of the collected data, especially as users of iOS devices are typically characterized by a high readiness to adopt novel digital solutions. Moreover, it does not limit the feasibility of real-world deployment, as the app architecture can be readily adapted for the Apple iOS platform. Nonetheless, reliance on smartphone-based voice recording may pose usability challenges for older patients or individuals with limited digital literacy. To mitigate this issue, the app was designed with a strong emphasis on simplicity and user-friendliness.

Patient selection for this study was limited to individuals with a high frequency of mental state changes in order to ensure sufficient data for training and evaluation purposes. This approach may limit the generalizability to a more stable population. Most of the limitations of future research work will gradually be eliminated as technology develops and broader populations of patients with mental disorders are included.

Considering the nature of validation and the request that each alert be assessed by a doctor, we decided to use only one machine learning model. Therefore, comparative performance between candidate models cannot be fully evaluated.

Due to the high variability between subjects and the complexity of the problem being considered, individualized thresholds are necessary.

The study’s naturalistic design involved clinicians recruiting patients from their own practices. During the evaluation phase, researchers were aware when contact with a patient was prompted by a system alert, which may have introduced confirmation bias. To mitigate this risk, the protocol mandated the use of standardized psychiatric tools, including the HDRS and the YMRS, along with structured questionnaires. These standardized procedures were also used to minimize incorporation bias. However, this still likely had the effect of inflating the perceived usefulness of the alert.

The Polish-only training sample was validated. It is expected that the acoustic feature-mood relationships will transfer across languages, but empirical verification is required for clear confirmation (at the time of this study’s publication, our team is currently conducting further research on a large group of Spanish-speaking patients).

### Comparison With Previous Work

The present findings confirm and extend previous evidence that acoustic features are robust biomarkers of mood states in individuals with BD, a line of research that historically began as early as the 1940 s and continued with the development of new technical capabilities [20,28,29,35].

Early studies demonstrated that changes in speech activity and voice characteristics are sensitive and reliable indicators of prodromal symptoms preceding affective episodes. This allows for early and objective quantification of psychopathological changes in patients [29,36,37]. Although speech was confirmed as a promising marker for mental health assessments by those early studies, there were still many technical obstacles to overcome in designing and implementing effective systems and reliable algorithms [38], and many further efforts were reported in this area. An example is a clinically validated digital mental health platform that integrates patient-reported outcomes and passive sensor data to support symptom monitoring, which was validated for many groups, including young patients [39]. However, advanced statistical and AI tools like pattern analysis and risk assessments are still limited to research purposes. Another large-scale European project that uses wearable devices and mobile technology to monitor and prevent relapses in BD, depression, and epilepsy is Remote Assessment of Disease and Relapse-Central Nervous System (RADAR-CNS) [40]. However, RADAR-CNS is mainly dedicated to remote data collection and efficient integration of various sensor data. In comparison, the MoodMon system focuses on advanced, AI-driven voice monitoring, tightly integrated with the combined processing of speech signals and psychiatric assessment. Another recent project is BEHAPP [41], which is a research platform for passive data collection through smartphones to support scientific and medical research. Depending on the study, BEHAPP may collect various types of data such as location, call and SMS text messaging history, Wi-Fi access points, app usage, motion through accelerometers, ambient light, and noise levels. Again, MoodMon goes beyond the current state of the art due to its AI toolbox tailored to psychiatrically guided voice analysis.

The key innovation of the MoodMon system lies in the use of AI to predict dynamic changes in mental state, whereas previous solutions relied primarily on the collection of objective but largely static data reflecting remission or the presence of an affective episode. Therefore, direct comparison of MoodMon with previously described systems for monitoring affective disorders is inherently difficult. Compared to previous solutions, the MoodMon system represents a methodological and technological advancement. Its longitudinal implementation has generated an exceptionally large dataset for training machine learning models. Previous studies have primarily focused on differentiating affective episodes from remissions in individuals with BD based on voice, demonstrating moderate accuracy compared to conventional mood scales (approximately 0.61‐0.74). These studies have highlighted the potential of speech-derived features for classifying affective states (depressive, manic, and euthymic) as a complement to established clinical tools, thereby improving monitoring of individuals in real-world settings [34,42].

It is important to emphasize that a critical element of patient safety in managing AI in the MoodMon system is constant human supervision by a qualified psychiatrist. The clinician is responsible for continuously assessing whether the AI is performing its function properly—namely, generating timely alerts in response to changes in the mental state of the patient. In this study, the frequency of such human assessments exceeded that reported in comparable studies, contributing to improved AI performance and accuracy.

The MoodMon system’s analytical framework is based on both individual longitudinal data and aggregated group data, resulting in significantly larger training datasets than those used in previous studies [20,30,43].

Furthermore, the study adhered to the latest recommendations for AI applications in psychiatry, including incorporating patient-reported subjective assessments of the MoodMon system [33]. Consistent with the findings of a recent Italian study examining the correlations between speech characteristics and clinical status in individuals with bipolar depression or mania, our results support the conclusion that acoustic features are an important complement to clinical practice in mobile health ecosystems [40,44].

The improved performance is achieved by limiting the classification to 2 classes, whereas previous works strived to classify the states of the patient into 3 or more classes [17,20,27]. Moreover, MoodMon used voice exclusively as input for detecting changes in mental state, while previous works [17,20] also used other behavioral markers and self-reported evaluations.

The naturalistic study gathered a patient population typical of groups treated in outpatient settings; however, detailed data, broken down by the psychopathological differences of both diagnoses, can be found in separate publications from the project [45,46]. Furthermore, in Kaczmarek-Majer et al [47], we propose additional explanations based on linguistic summaries and fuzzy rule-based reasoning models to support alerts generated by the system.

Clinical use of the MoodMon system in the monitoring and management of patients with affective disorders demonstrated that system-generated alerts constitute clinically meaningful support for decision-making. The presence of alerts was associated not only with an increased probability of patient-clinician contact but also with a significantly higher likelihood of therapeutic modification. This represents a key clinical implication, as it confirms that the primary objective underlying the interdisciplinary development of the MoodMon system—namely, to provide an effective, clinically actionable tool for both patients and psychiatrists—has been successfully achieved.

### Conclusions

The MoodMon study confirmed that acoustic features derived from speech serve as objective biomarkers of mental state changes in individuals with BD and MDD.

The high effectiveness achieved confirmed the validity of the approach based on the limitation of the classification to only 2 classes.

Alerts generated by the system effectively support psychiatrists in clinical decision-making while maintaining their responsibility for final diagnostic and therapeutic decisions.

The high efficiency and practicality of the presented AI system translate into a high potential for implementation in clinical practice as a modern, smartphone-based tool for continuous monitoring of patients with affective disorders.

## Abbreviations

BD: bipolar disorder
CGI: Clinical Global Impression Scale
FN: false negative
FP: false positive
HDRS: Hamilton Depression Rating Scale
MDD: major depressive disorder
RADAR-CNS: Remote Assessment of Disease and Relapse-Central Nervous System
TN: true negative
TNR: true negative rate
TP: true positive
TPR: true positive rate
YMRS: Young Mania Rating Scale
